# Body configuration at first stepping-foot contact predicts backward balance recovery capacity in people with chronic stroke

**DOI:** 10.1371/journal.pone.0192961

**Published:** 2018-02-22

**Authors:** Digna de Kam, Jolanda M. B. Roelofs, Alexander C. H. Geurts, Vivian Weerdesteyn

**Affiliations:** 1 Department of Rehabilitation, Donders Institute for Brain, Cognition and Behaviour, Radboud University Medical Center, Nijmegen, The Netherlands; 2 Sint Maartenskliniek Research, Nijmegen, The Netherlands; The Ohio State University, UNITED STATES

## Abstract

**Objective:**

To determine the predictive value of leg and trunk inclination angles at stepping-foot contact for the capacity to recover from a backward balance perturbation with a single step in people after stroke.

**Methods:**

Twenty-four chronic stroke survivors and 21 healthy controls were included in a cross-sectional study. We studied reactive stepping responses by subjecting participants to multidirectional stance perturbations at different intensities on a translating platform. In this paper we focus on backward perturbations. Participants were instructed to recover from the perturbations with maximally one step. A trial was classified as ‘success’ if balance was restored according to this instruction. We recorded full-body kinematics and computed: 1) body configuration parameters at first stepping-foot contact (leg and trunk inclination angles) and 2) spatiotemporal step parameters (step onset, step length, step duration and step velocity). We identified predictors of balance recovery capacity using a stepwise logistic regression. Perturbation intensity was also included as a predictor.

**Results:**

The model with spatiotemporal parameters (perturbation intensity, step length and step duration) could correctly classify 85% of the trials as success or fail (Nagelkerke R^2^ = 0.61). In the body configuration model (Nagelkerke R^2^ = 0.71), perturbation intensity and leg and trunk angles correctly classified the outcome of 86% of the recovery attempts. The goodness of fit was significantly higher for the body configuration model compared to the model with spatiotemporal variables (p<0.01). Participant group and stepping leg (paretic or non-paretic) did not significantly improve the explained variance of the final body configuration model.

**Conclusions:**

Body configuration at stepping-foot contact is a valid and clinically feasible indicator of backward fall risk in stroke survivors, given its potential to be derived from a single sagittal screenshot.

## Introduction

Falls are a considerable health problem after stroke because of their major physical and psychological consequences. Even in chronic stroke survivors, the risk of falls is substantially higher than in the general older population (1.4–5 vs 0.65 falls per year) [1]. Balance problems are among the most important risk factors for falls. Training programs aimed at improving balance effectively reduce the risk of falling in older individuals [2]. In people with stroke, however, similar type of exercise programs have failed to reduce fall risk [3]. To develop effective fall preventive strategies in people after stroke, better insight in critical determinants of falling is needed.

In daily life situations, reactive stepping after a loss of balance is an important saving strategy to prevent an actual fall. Such stepping responses are substantially impaired in people after stroke compared to healthy individuals [4–9]. When exposed to balance perturbations in a laboratory situation, people after stroke demonstrated later and smaller steps and their center of mass (COM) was closer to the boundaries of their base of support (BOS) at the moment of first foot contact [5, 8]. Deficits in step kinematics (i.e. excessive trunk flexion and impaired step length) were associated with a greater likelihood of perturbation-induced falls in the forward direction [10]. Yet, it remains to be investigated, which step characteristics are most critical for backward balance recovery in stroke survivors.

Studies in healthy individuals have already provided important insight into critical determinants of balance recovery capacity following backward perturbations. It was found that body configuration at the instant of first stepping-foot contact could discriminate between single step and multiple step balance recovery attempts in healthy older individuals [11]. In another study, healthy young individuals were exposed to large magnitude backward perturbations that resulted in actual falls in 42% of the trials [12]. Following these perturbations, vertical leg and trunk inclination angles at first stepping-foot contact could correctly classify 96% of the trials as a successful or a failed recovery attempt. Traditional spatiotemporal step parameters (step onset, step length and step duration) could only classify 84% of the attempts correctly, indicating that simple body configuration parameters at first stepping-foot contact are sufficient to quantify the quality of backward reactive steps in healthy individuals. Such parameters could be highly valuable to identify people that are prone to falling in high-risk populations, such as stroke. However, it needs to be investigated whether such a simple model also applies to a much more heterogeneous group of people with stroke, in whom neuromuscular functions like muscle strength and coordination can vary greatly between subjects and also between the paretic and non-paretic leg within subjects.

In the present study, we aimed to validate body configuration parameters as outcome measures for step quality in chronic stroke survivors. We first compared backward reactive stepping performance (i.e. ability to recover balance with a single step) between stroke survivors and healthy controls to ensure that the selected group of stroke survivors was impaired in their step quality. We then determined the strength of the associations between body configuration outcomes and spatiotemporal step parameters. Finally, we compared the predictive value of body configuration vs. spatiotemporal parameters for the capacity to recover with a single step from a backward perturbation in the total group of stroke survivors and controls. We further evaluated whether the step variables identified as the strongest determinants, could predict backward stepping performance independent of disease (i.e. stroke or control) and stepping leg (i.e. paretic or non-paretic). We hypothesized that both body configuration and spatiotemporal parameters would be predictive of balance recovery capacity (i.e. single or multiple step). Based on previous findings in healthy individuals [12], we expect body configuration parameters to have a greater predictive value than spatiotemporal parameters.

## Methods

### Participants

Twenty-four ambulatory people in the chronic phase (> 6 months) after a unilateral supratentorial stroke as well as 21 healthy older adults (aged > 55 years) participated in this study (Table 1). Participants had to be able to stand and walk independently or under supervision (Functional Ambulation Categories (FAC) ≥ 3). Individuals who suffered from neurological (except stroke), cognitive (Mini Mental State Examination (MMSE) < 24) or musculoskeletal impairments as well as people who used medication that affects reaction time (e.g. neuroleptics and benzodiazepines) were excluded. Written informed consent was obtained from all participants. The protocol was approved by the Medical Ethical Board of the region Arnhem-Nijmegen and all procedures were conducted in accordance with the Declaration of Helsinki.

**Table 1:** Participant’s characteristics.

	People with stroke (n = 24) Mean (SD) or number	Healthy controls (n = 21) Mean (SD) or number
Gender (male/ female)	19/5	6/15
Age (years)	61.1 (9.1)	64.3 (5.2)
Body Weight (kg)	82 (14)	71 (15)
Height (m)	1.73 (0.10)	1.69 (0.09)
Time since stroke (months)	60 (48)	NA
Paretic side (left/ right)	13/11	NA
Type of stroke (ischemic, hemorrhagic)	19/5	NA
Fugl-Meyer Assessment—leg score	28.4 (4.1)	NA
Motricity Index—leg score	75.0 (10.1)	NA
Berg Balance Score	52.0 (4.9)	55.9 (0.4)

NA = not applicable. Possible score ranges for the clinical tests are: Fugl-Meyer Assessment—leg score, 0–34; Motricity Index—leg score, 0–100; Berg Balance Score 0–56.

### Experimental setup

**Fig 1:**
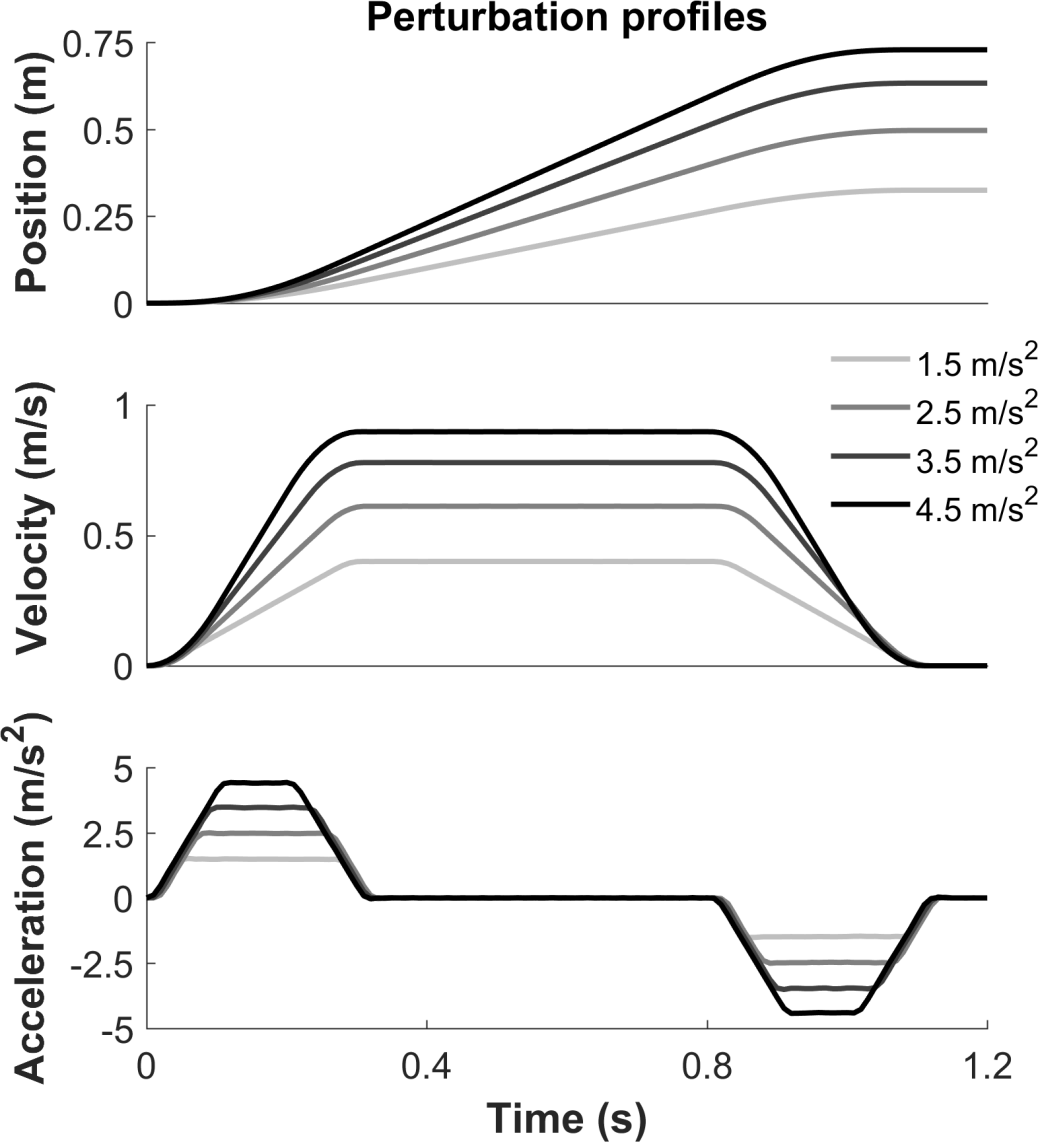
Perturbation profiles. Perturbation profiles for the different intensities of perturbation.

Participants stood barefoot with their feet 4.5 centimeters apart on a moveable platform (Length x Width: 240 cm x 174 cm [13]). The platform could unexpectedly translate in either of four directions (forward, backward, leftward and rightward). The perturbation waveform involved a 300 ms acceleration phase followed by a 500 ms constant velocity period and a 300 ms deceleration phase (See Fig 1 for perturbation profiles). Participants wore a safety harness that was attached to a sliding rail in the ceiling and which moved synchronically with the anteroposterior movements of the platform. People with stroke wore an ASO ankle brace (Medical Specialities, Wadesboro, North Carolina, USA) on the paretic side to prevent ankle injury.

### Experimental protocol

Participants were instructed to recover from the perturbations with a maximum of one step and not to grab the rail. A trial was classified as ‘success’ if there was no further backward extension of the base of support after the first step. After eight practice trials, the perturbation intensity was gradually increased until participants were unable to respond with a single step or until the maximum perturbation intensity was reached. Four trials were collected at each of four fixed intensities (1.5, 2.5, 3.5, 4.5 m/s^2^) as far as feasible for the participant. In order to minimize anticipation to the perturbations, backward perturbations were alternated with forward and sideways perturbations.

### Data collection and analysis

Full body kinematics (Vicon Plug-in-Gait) were recorded at 100 Hz using an 8-camera 3D motion capture system (Vicon Motion Systems, Oxford, UK). An additional reflective marker was placed on the translating platform to correct marker positions for platform movement. Marker trajectory data was filtered with a second order, 5 Hz low-pass, zero-lag Butterworth filter. The start of the perturbation was determined from a digital platform position signal and the instants of step onset and foot contact were determined from the foot marker recordings. Vertical leg and trunk inclination angles at foot contact were calculated in the sagittal plane. The trunk segment was defined by the line connecting the mid-shoulder to the mid-pelvis and the leg segment was defined by the line connecting the mid-pelvis to the 2^nd^ metatarsal of the stepping-foot. A forward tilted trunk and a foot position posterior to mid-pelvis were defined as a positive trunk and leg angle, respectively. All outcome measures were calculated with a custom written Matlab Program.

### Statistical analysis

To evaluate whether stroke survivors and controls differed in their capacity to sustain the increasing perturbation intensities, we conducted a survival analysis on the number of individuals that were still in the experiment at the different perturbation intensities.

To determine which step parameters were different between successful and failed recovery attempts, we used separate independent samples t-tests for each perturbation intensity. The strength of the relations between spatiotemporal step and body configuration parameters were quantified by Pearson correlations. To compare the predictive value of both body configuration and spatiotemporal step parameters for balance recovery capacity, we used a stepwise logistic regression analysis for each group of parameters separately. Perturbation intensity was also included as a possible predictor in each of the models. We used a forward stepwise logistic model with a likelihood ratio criterion. Variables were entered in the model if their p-value upon entry in the model was smaller than 0.05. Variables that were entered into the model were removed if their p-value changed to > 0.10 upon entry of subsequent predictors. Subsequently, we determined which of the two models (i.e. body configuration or spatiotemporal variables) better predicted the probability of successful balance recovery. This was done by comparing the models’ differences in the sum of the squared residuals (i.e. goodness of fit) to the differences in residuals that could be expected by chance under the null hypothesis of equal goodness of fit. To determine the distribution of the latter, we randomly flipped each individuals’ residual between the two models and again computed the difference of the sums of squares for the two models. We repeated this procedure 1 million times to generate a null distribution and determined the p-value based on this distribution. This analysis was performed in R studio.

## Results

### Successfulness of backward balance recovery attempts

A total of 522 trials with a backward perturbation were available for analysis, 235 of which were obtained from the people with stroke. Five participants in the stroke group failed in all backward trials. One participant with stroke (4%) succeeded in all trials, whereas this was true for two of the control subjects (10%). The remaining participants had both successful and failed recovery attempts.

**Fig 2:**
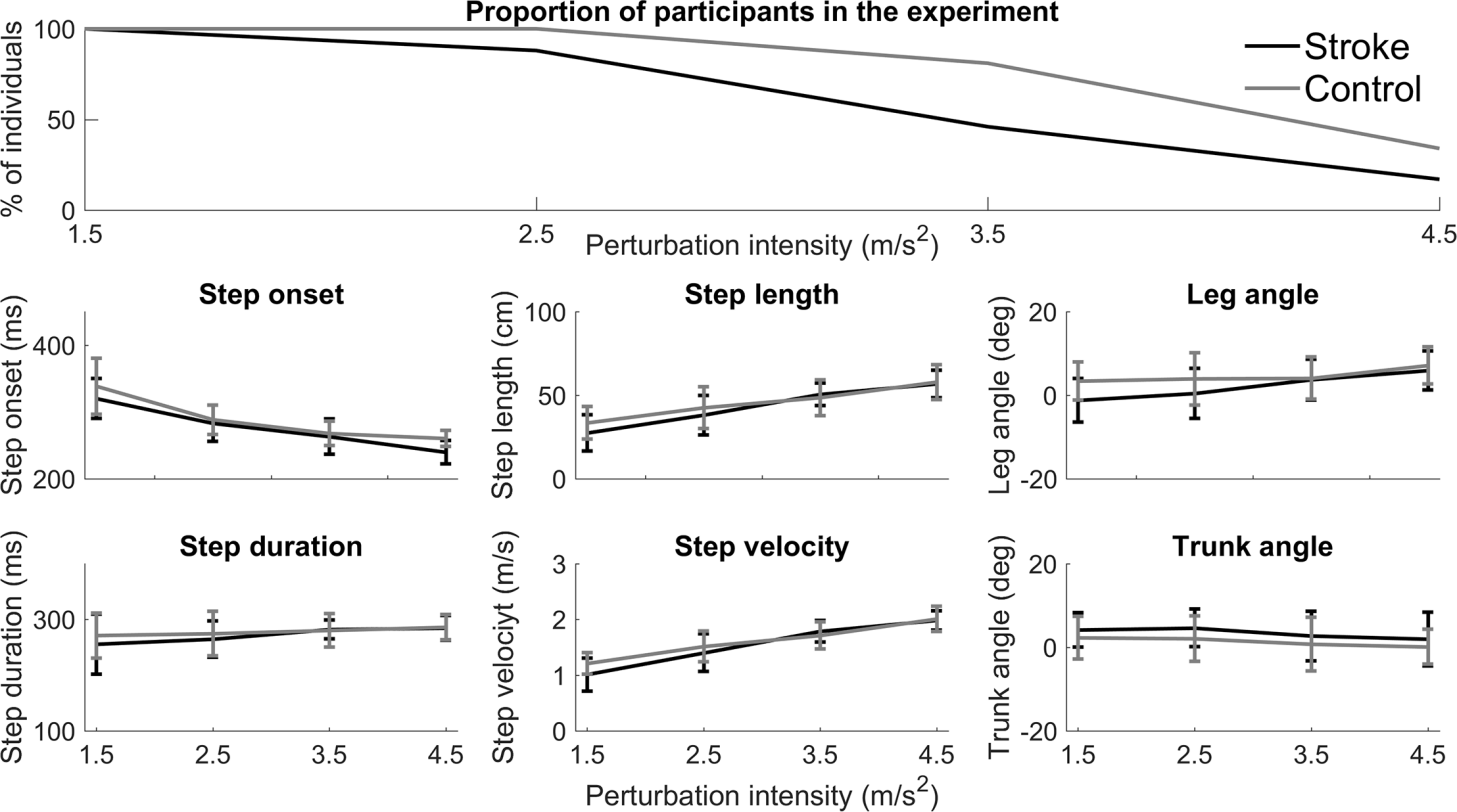
Descriptive information on stepping performance. Top panel: Proportion of participants that were still in the experiment at increasing perturbation intensities. The survival curve shows that the experiment was terminated at lower perturbation intensities in the people with stroke compared to the control subjects (chi^2^ = 4.6, p = 0.032), indicating that their balance recovery capacity was poorer. Lower panels: Descriptive data of spatiotemporal and body configuration parameters for each participant group at the four different perturbation intensities.

Participants remained in the experiment until they consistently failed to recover from the perturbations with a single backward step. As a consequence, 66% of the controls and 83% of the people with stroke had missing data at one or more of the fixed perturbation intensities. The survival curve in Fig 2 (top panel) shows that the experiment was terminated at lower perturbation intensities in the people with stroke compared to the control subjects (chi^2^ = 4.6, p = 0.032), indicating that their balance recovery capacity was poorer.

Descriptive data for both spatiotemporal and body configuration parameters are presented in Fig 2 (lower panels). As only individuals with better recovery capacity were tested at the higher perturbation intensities, step parameters were increasingly determined by those individuals. Comparison of step parameters between people with stroke and controls would thus suffer from selection bias. Therefore, we did not perform between-group statistics on the step parameters.

### Predictive value of body configuration and spatiotemporal step parameters

Table 2 presents body configuration and spatiotemporal step parameters for successful (single step) and failed recovery attempts. For all perturbation intensities, successful recovery attempts were characterized by more positive leg inclination angles, greater step length, higher step velocity, and longer step duration (p<0.01). We observed significant Pearson correlations between leg and trunk angles and most of the spatiotemporal step parameters (Table 3). Strong positive associations were found between leg angles and step length, duration and velocity (r>0.77, p<0.01). The same spatiotemporal parameters were weakly and negatively associated with trunk inclination angles (r<-0.23, p<0.01).

**Table 2:** Descriptive statistics for failed and successful attempts.

Perturbation intensity (m/s^2^)	Out-come	Number of trials	Leg angle (degrees)	Trunk angle (degrees)	Step onset (s)	Step Length (m)	Step Duration (s)	Step Velocity (m/s)
1.5	S	135	3.5 (4.5) #	3.1 (5.0)	0.33 (0.05)#	0.35 (0.09)#	0.28 (0.05)#	1.23 (0.19)#
	F	55	-6.2 (3.6)	4.7 (5.5)	0.31 (0.03)	0.16 (0.08)	0.21 (0.05)	0.74 (0.27)
2.5	S	94	6.5 (4.2)#	2.5 (5.3)#	0.29 (0.03)	0.48 (0.09)#	0.29 (0.03)#	1.66 (0.17)#
	F	69	-3.4 (5.1)	4.8 (5.2)	0.29 (0.03)	0.30 (0.10)	0.24 (0.03)	1.20 (0.31)
3.5	S	51	7.9 (3.4)#	1.7 (4.6)	0.26 (0.02)	0.56 (0.08)#	0.29 (0.02)#	1.92 (0.18)#
	F	58	0.9 (4.0)	1.2 (7.5)	0.27 (0.07)	0.44 (0.08)	0.27 (0.03)	1.60 (0.19)
4.5	S	39	9.0 (3.6)#	-0.6 (4.3)*	0.25 (0.01)	0.62 (0.09)#	0.30 (0.02)#	2.08 (0.20)#
	F	21	2.7 (4.5)	2.8 (5.7)	0.25 (0.03)	0.50 (0.08)	0.27 (0.02)	1.84 (0.18)

*p<0.05

#p<0.01 for difference between successful and failed attempts. S = success, F = fail.

**Table 3:** Correlation between body configuration and spatiotemporal parameters.

	Step onset	Step length	Step duration	Step velocity
**Leg angle**	-0.18#	0.88#	0.78#	0.81#
**Trunk angle**	0.08	-0.27#	-0.24#	-0.26#

#p<0.01 for Pearson correlation

Table 4 shows the results of the stepwise logistic regression analyses. For the body configuration model, perturbation intensity, leg angle, and trunk angle were retained in the final model. Together, those parameters explained 71% of the variance in recovery outcome and a total of 448 of the 522 trials (86%) were correctly classified as success or fail. For the model with spatiotemporal step parameters, 61% of the variance in balance recovery capacity could be explained by perturbation intensity, step length, and step duration. With these predictors, a total of 445 of the 522 trials (85%) was correctly classified as success or fail. The sum of squared residuals was lower for the body configuration model compared to the model with spatiotemporal variables. The permutation test revealed that this difference was larger than expected by chance (p<0.01 for comparison with the null-distribution computed as described before), indicating that the body configuration model was superior in predicting the probability. With this model, the probability of successful single stepping balance recovery could be quantified with the following equation:

$$\mathrm{Probability}\;\mathrm{of}\;\mathrm{success}=1‑(\frac{1}{1+e^{4.03-1.86*perturbation\;intensity+0.64*leg\;angle+0.06*trunk\;angle}})$$

**Table 4:** Results of the stepwise regression analyses.

	Mean (sd)	Odds ratio (95% CI)	p
***Body configuration parameters (Nagelkerke R***^***2***^ ***= 0*.*71*, *85*.*8% of trials correctly classified***)			
Perturbation intensity (m/s^2^)	2.6 (1.0)	0.16 (0.10–0.24)	<0.001
Leg angle (degrees)	2.6 (6.3)	1.90 (1.69–2.12)	<0.001
Trunk angle (degrees)	2.7 (5.6)	1.06 (1.00–1.12)	0.037
***Spatiotemporal parameters (Nagelkerke R***^***2***^ ***= 0*.*61*, *85*.*2% of trials correctly classified***)			
Perturbation intensity (m/s^2^)	2.6 (1.0)	0.04 (0.02–0.08)	<0.001
Step length (cm)	40 (15)	1.36 (1.27–1.46)	<0.001
Step duration (ms)	270 (44)	0.98 (0.96–0.99)	0.002

**Fig 3:**
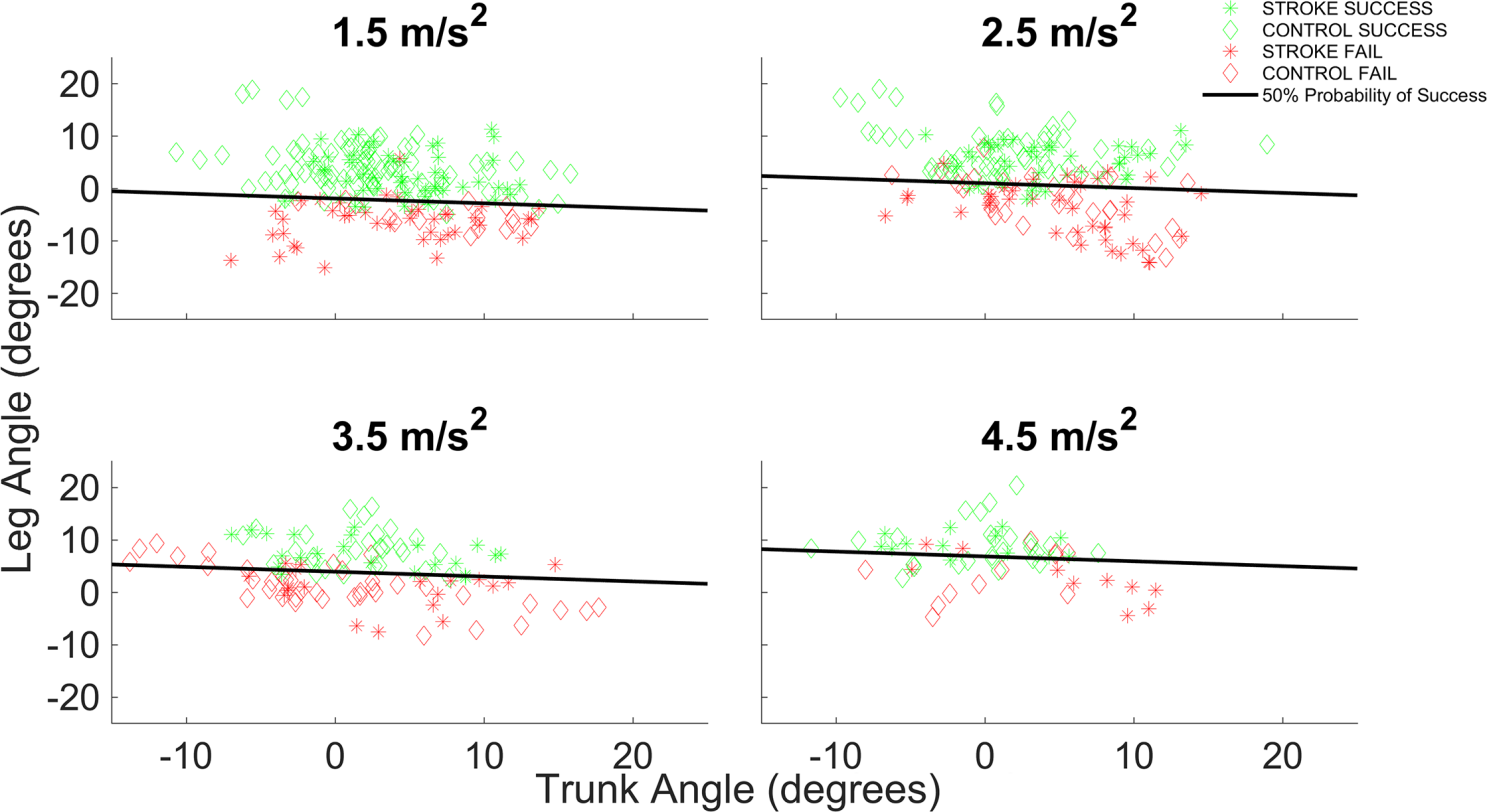
Predictive value of leg and trunk angles. Leg and trunk inclination angles for failed and successful recovery attempts at the different perturbation intensities. Solid lines represent the values corresponding to a 50% probability of success as determined by the logistic regression analysis.

Fig 3 demonstrates that there was very little overlap in particularly the leg inclination angles between failed and successful balance recovery attempts, indicating that leg inclination angle is a stronger predictor of backward balance recovery capacity than the trunk angle. Indeed, a 1° increase in leg angle increased the odds of successful balance recovery by almost twofold (OR = 1.9), which was equivalent to a 10.7° change in trunk angle. The regression lines further demonstrated that the leg angles corresponding to a 50% probability of success increased with perturbation intensity, which indicates that larger (i.e. better) leg inclination angles are required to successfully recover balance at greater perturbation intensities.

Overall, people in the control group were more likely to successfully recover balance with a single step than were people with stroke (67% vs. 54%, OR = 1.9, p<0.01). Yet, when we entered participant group as a predictor to the body configuration model it was not retained during the stepwise procedure, which implies that the model’s predictive ability was independent of the presence of stroke. Similarly, given the differences in gender distribution between people with stroke and controls, we also checked if this may have biased the regression analysis. This was not the case as gender was not retained in the final model when added to the stepwise regression analysis.

In our group of stroke survivors, the non-paretic leg was used for the first reactive step in 71% of the trials. Non-paretic stepping was associated with a greater likelihood of single step balance recovery compared to paretic stepping (60% vs. 38%, OR = 2.5, p<0.01). We therefore also determined whether body configuration could predict balance recovery capacity, regardless of stepping leg (paretic/non-paretic). In an additional logistic regression analysis within the stroke group, stepping leg also failed to survive the stepwise procedure.

## Discussion

We aimed to determine whether body configuration at first stepping-foot contact could predict balance recovery capacity following backward perturbations in chronic stroke survivors. As hypothesized, leg and trunk inclination angles at stepping-foot contact were stronger determinants of single-step balance recovery than spatiotemporal step parameters. A foot position more posterior to the pelvis and a more forward tilted trunk were associated with a greater likelihood of successful single step balance recovery, together explaining as much as 71% of balance recovery capacity at a given perturbation intensity.

People in the stroke group were less likely than controls to successfully recover from backward perturbations with a single step. Yet, participant group did not significantly add to the explained variance in recovery capacity when entered in the body configuration model. Similarly, in our group of stroke survivors, stepping leg did not add explained variance either, despite significantly poorer success rates for paretic vs. non-paretic steps. Hence, the poorer success rates in people after stroke, particularly when stepping with the paretic leg, can be accounted for by a less favorable body configuration at stepping-foot contact.

The findings of this study raise the question as to why people after stroke achieve less favorable body configurations at stepping-foot contact. The ability to make a sufficiently long backward step seems important, since leg angles were most strongly associated with step length (r = 0.88). Yet, our as well as previous findings consistently demonstrate that the leg angle at first stepping-foot contact is a stronger predictor of backward balance recovery capacity [12]. We suggest that the leg angle outperforms step length for quantifying reactive step quality, because it does not only provide information about foot displacement, but also captures the (horizontal) distance between the COM relative to the posterior edge of the BOS [14, 15]. Impaired backward balance recovery in people after stroke can thus be explained by a poorer ability to place the stepping leg far enough behind the COM [5].

In agreement with Weerdesteyn et al. [12] we found that the leg inclination angle at first stepping-foot contact was a much stronger predictor of backward balance recovery than the trunk angle (OR 1.9 vs. 1.06). In addition, it must be pointed out that trunk angle has hardly any predictive value with regard to balance recovery capacity when leg angle is not taken into account (Table 2). The observation that the trunk angle plays at most a minor role in the ability to recover from backward perturbations contrasts with previous studies demonstrating a more crucial role of trunk kinematics to restore balance after forward perturbations. [10, 16, 17]. More specifically, the ability to resist forward trunk flexion appeared to be critical to prevent falling following forward perturbations in both healthy individuals [17] and stroke survivors [10]. We suggest that, for backward perturbations, a similar mechanism (i.e. resisting backward tilting of the trunk) plays a less important role, because the anatomical range of motion of the trunk is much smaller for extension compared to flexion movements.

Another factor that has shown to play an important role in overcoming forward perturbations is the use of eccentric knee extensor torques to resist further COM displacement after stepping-foot contact [18]. Yet, for backward balance recovery, the previous observation that body configuration at first stepping-foot contact almost perfectly predicted whether healthy individuals would eventually fall following very large balance perturbations argues against a major role of post-landing joint torques [12]. Our finding that body configuration predicts balance recovery capacity regardless of stepping leg (paretic / non-paretic / control) further supports the idea of a minor influence of post-landing joint torques. If such torques would be critical for successful balance recovery, differences in muscle strength between stroke survivors and controls would probably have resulted in group being an independent predictor of success in addition to body configuration.

While the vast majority of the recovery attempts could be correctly classified as success or failed, about 14% of the trials was misclassified. Importantly, in most of the misclassified trials the leg and trunk angles were very close to the critical values determined by the regression model (Fig 3). In some other trials, participants took more than one step, despite good leg and trunk angles. Such false positive observations may be explained by participants’ fear of falling backward, which made them take an extra backward step out of cautiousness. Lastly, it is worth pointing out that very poor leg and trunk angles never resulted in successful balance recovery, which observation further supports the notion that a good-quality first step is critical for successful balance recovery.

### Clinical implications

Insight in key determinants of successful balance recovery is crucial for identifying stroke survivors at risk of falling. Previous studies have identified stroke-related deficits in reactive step kinematics as well as determinants of successfulness of balance recovery in people after stroke [4–6, 8, 10, 19]. Our findings add to these previous observations by identifying a set of key parameters that are most critical for successful balance recovery following backward balance perturbations. These body configuration parameters (i.e., leg and trunk angle) capture the COM-BOS relationship at first stepping-foot contact and are much easier to implement in a clinical testing paradigm than COM based measures, as they can potentially be derived from a ‘sagittal screenshot’ at first foot contact. It is important for these testing paradigms to standardize the intensity of the perturbations, given our observation that the required leg and trunk angles to recover balance depend on perturbation intensity. We suggest that relatively low perturbation intensities will most likely be sufficient to identify people at risk of falls, since stroke survivors who fall at higher perturbation intensities already demonstrate poorer steps at lower intensities [10]. This allows for the development of testing protocols that are safe and feasible for a broad population of stroke survivors.

### Study limitations

A limitation of this study was that, for safety reasons, we did not expose our participants with stroke to perturbation intensities at which they would actually fall. We therefore defined the use of single vs. multiple steps as an alternative criterion for balance recovery capacity. Although we instructed participants to try their hardest to recover balance with a single step, some individuals may have taken more steps, even if not strictly necessary for balance recovery. This may explain why the association between body configuration parameters and balance recovery capacity was not as strong as in a previous study that exposed young individuals to highly destabilizing perturbations [12]. Yet, this previous study also demonstrated that, after large perturbations, the quality of the first step is most critical for balance recovery, which justifies the use of single vs. multiple stepping as a proxy indicator of balance capacity. In addition, individuals who fall at high perturbation intensities also demonstrate poor step quality at small perturbation intensities [10]. Hence, measuring leg and trunk angles at foot contact of the first balance correcting step appears to be a feasible and valid method for assessing backward reactive step quality in people with chronic stroke.

A second limitation is that there may have been a learning or habituation effect throughout the experiment. Indeed, in the paper of Weerdesteyn and coworkers it was found that in healthy young adults, the probability of successful recovery increased with repeated perturbations [12]. Yet, these improvements were found along with gains in leg angles, such that the association between body configuration and balance recovery outcome remained unchanged. Hence, the greater probability of success through learning or habituation appears to be mediated by a better execution of the first recovery step.

The final limitation of our study was that our sample was restricted to community dwelling chronic stroke survivors. It remains to be investigated whether body configuration is a good measure of step quality for people in the sub-acute phase after stroke and in people with more limited gait capacity. Our prediction is that leg angle remains an important determinant in these more affected populations, given our observation that our final model could predict balance recovery capacity regardless of group (stroke / control) and regardless of which leg was used for stepping. It is possible, however, that post-landing joint torques play a more important role as well, particularly in individuals who have difficulties bearing their full weight on the paretic leg.

## Conclusion

We demonstrated that body configuration at first stepping-foot contact can predict the capacity to recover from a backward balance perturbation with a single step in individuals with stroke. Leg and trunk angles at the instant of foot contact hold promise as clinically feasible parameters to identify individuals with stroke at risk of backward falls, given their potential to be derived from a single sagittal screenshot.

## Supporting information

S1 File Individual trial data. Data of individual trials used in the analyses.
(XLSX)
